# Impact of screening on cervical cancer incidence: A population‐based case–control study in the United States

**DOI:** 10.1002/ijc.32826

**Published:** 2019-12-31

**Authors:** Rebecca Landy, Peter D. Sasieni, Christopher Mathews, Charles L. Wiggins, Michael Robertson, Yolanda J. McDonald, Daniel W. Goldberg, Isabel C. Scarinci, Jack Cuzick, Cosette M. Wheeler, for the New Mexico HPV Pap Registry Steering Committee

**Affiliations:** ^1^ Wolfson Institute of Preventive Medicine Queen Mary University of London London United Kingdom; ^2^ Division of Cancer Epidemiology and Genetics, National Cancer Institute, National Institutes of Health, Department of Health and Human Services Bethesda MD; ^3^ School of Cancer & Pharmaceutical Sciences King's College London London United Kingdom; ^4^ Department of Internal Medicine University of New Mexico Comprehensive Cancer Center and University of New Mexico Health Sciences Center Albuquerque NM; ^5^ The Center for HPV Prevention University of New Mexico Comprehensive Cancer Center, University of New Mexico Health Sciences Center Albuquerque NM; ^6^ Department of Human and Organizational Development Vanderbilt University Nashville TN; ^7^ Department of Geography Texas A&M University College Station TX; ^8^ Division of Preventive Medicine University of Alabama at Birmingham Birmingham AL; ^9^ Department of Pathology and Obstetrics & Gynecology University of New Mexico Health Sciences Center Albuquerque NM

**Keywords:** cervical screening, cervical cancer, cytology, pap smear, HPV, case–control, cancer screening, cancer registry

## Abstract

Cervical cancer is widely preventable through screening, but little is known about the duration of protection offered by a negative screen in North America. A case–control study was conducted with records from population‐based registries in New Mexico. Cases were women diagnosed with cervical cancer in 2006–2016, obtained from the Tumor Registry. Five controls per case from the New Mexico HPV Pap Registry were matched to cases by sex, age and place of residence. Dates and results of all cervical screening and diagnostic tests since 2006 were identified from the pap registry. We estimated the odds ratio of nonlocalized (Stage II+) and localized (Stage I) cervical cancer associated with attending screening in the 3 years prior to case‐diagnosis compared to women not screened in 5 years. Of 876 cases, 527 were aged 25–64 years with ≥3 years of potential screening data. Only 38% of cases and 61% of controls attended screening in a 3‐year period. Women screened in the 3 years prior to diagnosis had 83% lower risk of nonlocalized cancer (odds ratio [OR] = 0.17, 95% CI: 0.12–0.24) and 48% lower odds of localized cancer (OR = 0.52, 95% CI: 0.38–0.72), compared to women not screened in the 5 years prior to diagnosis. Women remained at low risk of nonlocalized cancer for 3.5–5 years after a negative screen compared to women with no negative screens in the 5 years prior to diagnosis. Routine cervical screening is effective at preventing localized and nonlocalized cervical cancers; 3 yearly screening prevents 83% of nonlocalized cancers, with no additional benefit of more frequent screening. Increasing screening coverage remains essential to further reduce cervical cancer incidence.

## Abstract 2

**What's new?**

Screening is an effective means of preventing cervical cancer in women. However, while a negative screening result affords protection against cervical cancer, little is known about how long this protection lasts or when a woman should be screened again. Here, the authors estimate the impact of cervical screening at a state‐wide level, linking a U.S. population‐based screening registry with a SEER cancer registry. The results show that screening once every three years prevents 83 percent of stage 2+ cervical cancers, with no additional benefit from more frequent screening. The findings may help improve adherence to U.S. screening guidelines.

## Introduction

Cervical cancer is largely preventable, yet an estimated 13,170 women in the United States (US) will be diagnosed with invasive cervical cancer in 2019, an age‐standardized rate of 7.6 per 100,000 women in 2011–2016.^1^ Cervical screening and human papillomavirus (HPV) vaccination are two methods of preventing cervical cancer. In 2012, consensus guidelines were issued for cervical screening in US populations, recommending screening begin at age 21 years; 3 yearly cytology for women aged 21–29 years, and either 3 yearly cytology or 5 yearly cotesting (co‐occurring HPV and cytology testing) for women 30–64 years.^2^, ^3^ In 2018, the US Preventive Services Task Force (USPSTF) released updated guidelines, adding 5 yearly primary HPV testing as an option for women aged 30–65 years.^4^ Most women aged >65 years can cease cervical screening.^2^, ^4^ The first HPV vaccine was licensed in the US in 2006^5^ and the Centers for Disease Control and Prevention first recommended routine HPV vaccination for girls aged 11–12 years in 2007.^6^

Screening has been shown to be effective at preventing cervical cancer on a population level since the 1960s.^7^ Although the effectiveness of screening has been evaluated in numerous European populations,^7^, ^8^, ^9^, ^10^, ^11^, ^12^, ^13^ the sensitivity of cytology varies between screening settings.^14^ Previous research on the effectiveness of cervical screening within the US has focused on women enrolled in health plans or integrated health systems,^15^, ^16^ and/or has focused on women of specific ages.^17^ In 2006, HPV was added to the list of reportable conditions for individuals residing in New Mexico. All cervical screening test results (HPV, Pap cytology and cotesting) and all pathology for the cervix, vagina and vulva are reported to the New Mexico HPV Pap Registry (NMHPVPR).^18^ The NMHPVPR has previously been described in detail.^19^ New Mexico is the only State in the US with a complete record of all cervical screening, diagnosis and treatment, providing appropriate high‐quality data to evaluate the effectiveness of cervical screening on a population basis, across a variety of diverse healthcare delivery settings and populations. The population of New Mexico is diverse; according to 2018 population estimates, 49.1% of the population were of Hispanic or Latino origin, 10.9% were American Indian or Alaska natives and 2.6% were African American.^20^

We assessed the effectiveness of cervical screening in New Mexico using a case–control study design. We addressed three questions (outlined in the Methods) which together provide insights into the effectiveness of screening on a state‐wide basis. Our study was approved by the University of New Mexico Human Research Review Committee.

## Methods

### Cervical cancer cases

We collected data on all cervical cancer diagnoses in the population‐based New Mexico Tumor Registry (NMTR) during 2006–2016. For each case, the NMTR provided information on the month/year of birth, month/year of diagnosis, morphology and stage at diagnosis (using the derived AJCC‐6 stage classification system). NMTR records were linked with the NMHPVPR to provide information on each case's history of cervical screening, diagnostic and treatment results within New Mexico since January 2006. The reason why each test was performed was not available; see Supporting Information S1 for details on how we determined which tests were likely due to symptoms. Only colposcopy procedures resulting in a biopsy were captured. With few exceptions, information was available for each woman's census tract of residence at cancer diagnosis and at each screening or diagnostic test.

Since cancers histologically diagnosed within 5 months of an abnormal screening result were almost certainly present at the time of the screen, and in most cases will have been screen‐detected, we took the date of the first abnormal cytology or positive HPV test within 5 months of histological diagnosis as the “date of index diagnosis”. The date of index diagnosis for cases with no such abnormal test result was the date of diagnosis. We note that this definition primarily affects results when considering “time since last screen” since this definition does not count a positive test less than 5 months before histological diagnosis as a prediagnostic test.

### Controls

Controls were selected from the NMHPVPR. Five women were selected per case, matched on date of birth and census tract of residence at diagnosis. To be eligible as a control, women had to be alive without a known hysterectomy or diagnosis of cervical cancer recorded at the date of the case's diagnosis. Since women were only in the NMHPVPR if they had attended screening from January 2006–December 2016, we added a fractional number of unscreened “virtual‐controls” for each case, to represent women who had not attended screening between January 2006–December 2016, and were therefore not in the NMHPVPR. The number of virtual controls was determined by comparing numbers of women in NMHPVPR with numbers from the census. Details on how the weights were calculated to determine the fractional number of unscreened women are available in Supporting Information S2, and additional details on matching in Supporting Information S3. All controls were assigned their matched case's date of diagnosis as a date of pseudodiagnosis.

### Measures to evaluate the effectiveness of cervical screening

We address the following primary questions:

1. What is the risk of (*i*) Stage I (localized) and (*ii*) Stage II+ (nonlocalized) cervical cancer within 3 years of attending screening compared to the risk in women who did not attend screening within the previous 5 years?
2. For how long do women remain at lower risk of nonlocalized cancer after a negative screen?
3. How does the risk among women who attend screening frequently (at least once every 2.5 years), regardless of the screening result, compare with the risk among women who do not attend screening or who attend infrequently?

We examined the effect of attending screening on the risk of cervical cancer using the following measures to answer each question. (*i*) Existence of a satisfactory screen in the 3 years prior (*vs*. none in the 5 years prior) to the case's date of index diagnosis. This analysis was restricted to women with ≥3 years of potential prediagnosis screening history. (*ii*) Time between the last negative screening test and the case's date of index diagnosis, among women with ≥5 years of screening history available. A screening test was defined to be negative if there was a negative cytology or HPV test which was not taken as part of a positive cotest, nor was it the first negative cytology/HPV test within 12 months of an abnormal screening test. We used the following categories: ≤1.5, 1.5–2.5, 2.5–3.5 and 3.5–5 years, compared to women with no recorded negative screening tests within 5 years of the case's date of index diagnosis. (*iii*) We defined a woman to have been frequently screened if she had at least two screens a minimum of 10 months apart, with no interval >30 months between screens, in the 5 years prior to the date of index diagnosis/pseudodiagnosis. Women with some screening in the 5 years prior to the date of index diagnosis who did not meet the criteria of frequent screening were considered to have attended screening infrequently. This analysis was restricted to women with at least 5 years of screening history, to allow us to distinguish unscreened from infrequently screened women.

Since women are only recommended to attend routine screening until age 65 years, we restrict the main analyses to women aged 25–64 years. Except where explicitly stated otherwise, when analyses considered screening in a 5‐year period, we excluded cases and their matched controls diagnosed before January 1, 2011. All analyses were carried out for all stages combined and separately by stage at diagnosis.

We carried out seven sensitivity analyses (SA) on the first question addressed (What is the risk of (*i*) Stage I (localized) and (*ii*) Stage II+ (nonlocalized) cervical cancer within 3 years of attending screening compared to women who did not attend screening within the previous 5 years?). The first sensitivity analyses (SA1) adjusted for the census‐tract level sociodemographic variables shown in Supporting Information Table S1, since the controls were matched to the cases on census tract, and we do not have individual‐level sociodemographic data. SA2 excluded women whose address was a P.O. Box or zip code (Supporting Information S2). SA3 used an alternative set of weights, where control women from the NMTR who were diagnosed with potentially screen‐detected cancers (breast or colorectal) were excluded when calculating the weights. SA4 excluded the virtual (unscreened) controls from the analysis, to examine the impact of merely selecting controls from the NMHPVPR, without allowing for the fact that it is not a population register, and that women who did not attend screening from January 2006 to December 2016 could not be selected as a control. SA5 included women of all ages, regardless of whether they were recommended to attend screening, and SA6 included women aged 25–69 years, since 65 years was only introduced as the upper age limit of screening in 2012.^3^ Finally, SA7 used a reference category of women who had not attended screening in a 3‐year period, rather than a 5‐year period.

### Statistical methods

We present results from unadjusted weighted logistic regression analyses (having broken the matching, to allow for the weights) as the primary results.

Reporting under state regulations (New Mexico Administrative Code) specified by the list of Notifiable Diseases and Conditions is exempted from informed consent.

Data availability: Primary data supporting the investigation reported in this article can be made available in de‐identified form subject to establishing a data use agreement with the University of New Mexico Health Sciences Center.

## Results

**Figure 1:**
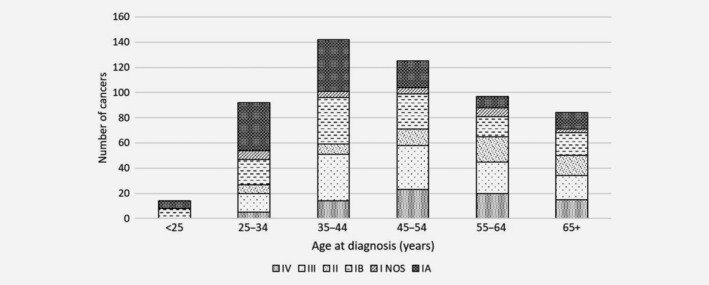
Stage distribution by age of the 646 cervical cancers diagnosed in New Mexico 2009–2016 among women with ≥3 years of screening history.

A total of 876 women were diagnosed with cervical cancer in New Mexico between January 1, 2006 and December 31, 2016. Of these 876 cancers, 70% were squamous, 19% adenocarcinoma, 2% adenosquamous and 8% other morphologies. A total of 646 women were diagnosed from January 2009–December 2016, with ≥3 years of potential screening history recorded. Of these, 47.9% were diagnosed at ages 35–54 years, with only 2.3% (*n* = 15) diagnosed before age 25 years, and 15.8% (*n* = 102) diagnosed aged ≥65 years (Fig. 1, Table 1). The stage at diagnosis was strongly related to age at diagnosis; in women <35 years, 75.0% with a known stage were Stage I, compared to 41.1% among women aged ≥65 years.

**Table 1:** Stage distribution by age of the 646 cervical cancers diagnosed in New Mexico 2009–2016 among women with ≥3 years of screening history

Age (years)	IA		I NOS		IB		II		III		IV		Unknown		Total	
	*n*	% of known stage	*n*	% of known stage	*n*	% of known stage	*n*	% of known stage	*n*	% of known stage	*n*	% of known stage	*n*	% of total	*n*	%
<25	6	42.9	0	0.0	8	57.1	0	0.0	0	0.0	0	0.0	1	6.7	15	2.3
25–34	39	41.5	8	8.5	20	21.3	7	7.4	15	16.0	5	5.3	10	9.6	104	16.1
35–44	45	30.4	6	4.1	38	25.7	8	5.4	37	25.0	14	9.5	14	8.6	162	25.1
45–54	22	16.7	5	3.8	29	22.0	14	10.6	37	28.0	25	18.9	15	10.2	147	22.8
55–64	9	8.7	7	6.8	19	18.4	22	21.4	26	25.2	20	19.4	13	11.2	116	18.0
65+	13	14.4	5	5.6	19	21.1	16	17.8	20	22.2	17	18.9	12	11.8	102	15.8
Total	134	23.1	31	5.3	133	22.9	67	11.5	135	23.2	81	13.9	65	10.1	646	100

NOS: not otherwise specified.

**Figure 2:**
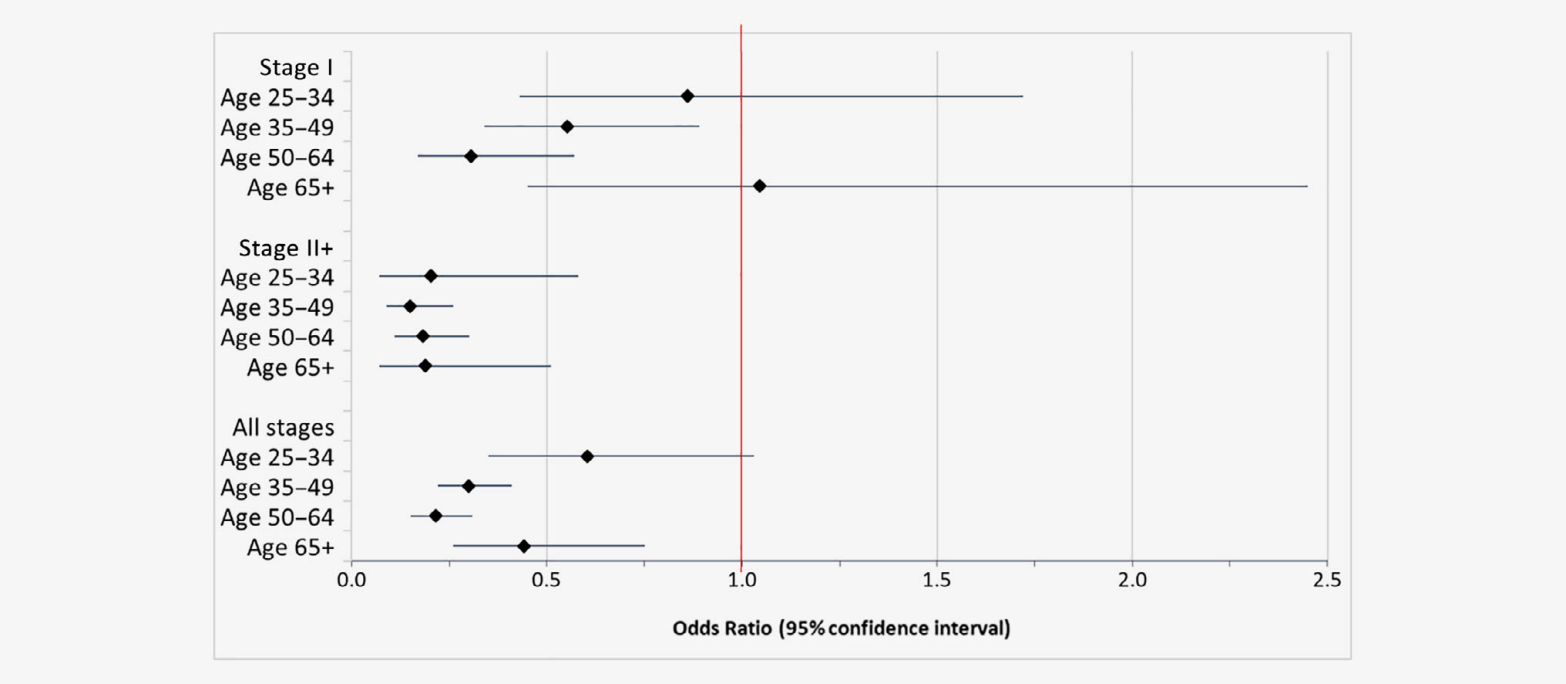
Odds ratios and 95% confidence intervals for risk of cervical cancer by stage for women screened within the last 3 years compared to women not screened in the last 5 years, restricted to women with ≥3 years of screening history. [Color figure can be viewed at wileyonlinelibrary.com]

Approximately 40% (38.0%) of cases diagnosed aged 25–64 years attended screening in the 3 years prior to the date of index diagnosis (Table 2), compared to 61.2% of controls (weighted for women without a record of screening in the NMHPVPR). Women aged 25–64 years who attended screening in a 3‐year period had a lower risk of diagnosis for each cancer stage compared to women not screened in the last 5 years (Table 2, Supporting Information Table S2). Only one‐fifth (22.5%) of women with Stage III+ cancer had been screened in the 3 years prior to the date of index diagnosis, compared to 59.3% of women with Stage IA cancer (Supporting Information Table S2). The effect of attending screening in the last 3 years increased with increasing cancer stage, from no effect on the odds of Stage IA cancer (odds ratio (OR) = 0.78, 95% CI:0.48–1.28) to strong effects on Stage III+ cancer (OR = 0.16, 95% CI:0.10–0.23) compared to women who did not attend screening in the last 5 years. Figure 2 shows there were statistically significant effects of screening on nonlocalized cancers for all ages, but only for ages 35–49 years and 50–64 years for Stage I cancers.

**Table 2:** Odds ratios and 95% confidence intervals of cervical cancer by screening attendance and stage at diagnosis, among women aged 25–64 years with at least 3 years of potential screening history

	Cases		Controls		OR (95% CI)	OR (95% CI)
	*n*	%	*n*	%		
Stage I						
Screened in the last 3 years	128	51.4	786.3	63.1	0.52 (0.38–0.72)	1
Screened in the last 5 years, but not the last 3 years^1^	53	21.3	242.5	19.4	0.70 (0.47–1.04)	1.34 (0.95–1.90)
Not screened in the last 5 years, with ≥5 years of potential screening data	68	27.3	217.9	17.5	1	1.92 (1.39–2.65)
Stage II+						
Screened in the last 3 years	57	24.9	680.8	60.1	0.17 (0.12–0.24)	1
Screened in the last 5 years, but not the last 3 years^1^	54	23.6	211.2	18.6	0.52 (0.36–0.75)	3.05 (2.05–4.55)
Not screened in the last 5 years, with ≥5 years of potential screening data	118	51.5	241.3	21.3	1	5.84 (4.14–8.24)
All Stages						
Screened in the last 3 years	200	38.0	1,610.3	61.2	0.30 (0.24–0.38)	1
Screened in the last 5 years, but not the last 3 years^1^	120	22.8	513.4	19.5	0.57 (0.44–0.73)	1.88 (1.47–2.40)
Not screened in the last 5 years, with ≥5 years of potential screening data	207	39.3	505.7	19.2	1	3.30 (2.66–4.08)

NMHPVPR and virtual controls were used in this analysis.

1 or not screened in the last 3 years with <5 years of potential screening data.

The results from SA are presented in Supporting Information Figure S1. Most of the SA provided extremely similar results, more details are provided in Supporting Information S4. When we assumed that the population at risk of cervical cancer excluded women with a hysterectomy (who guidelines have recommended against screening since 2012^2^), and that all hysterectomized women had not attended screening, the proportion of unscreened women was 0 for women aged 20–69. This is equivalent to SA4, when the virtual‐controls were excluded from the analyses; this SA showed a larger effect of screening (SA4).

For time since the last negative screen (Table 3), when restricted to women with ≥5 years of potential screening history, women aged 25–64 years with a negative screen remained at lower risk of both Stage I (OR = 0.20, 95% CI: 0.14–0.28) and nonlocalized cancer (OR = 0.11, 95% CI: 0.07–0.17) for at least 3.5 years compared to women with no negative screening in the last 5 years (a mix of women with no screening and those with only abnormal screening results). The risk for Stage II+ cancers remained constant over the first 3.5 years. Results were similar in the SA, adjusting for census‐level socioeconomic variables, and using alternative weights (Supporting Information Table S3). There was a significant reduction in risk of nonlocalized cervical cancer for at least 3.5 years following a negative test relative to women with no negative tests in 5 years for women in each age group considered (25–34, 35–49, 50–64 and ≥65 years), except Stage I for women aged ≥65 years (Supporting Information Fig. S2). In SA, when the analysis was extended to women with ≥3 years potential screening history rather than 5 years, the results were very similar (Supporting Information Table S4).

**Table 3:** Odds ratios and 95% confidence intervals of cervical cancer by time since last negative screen and stage at diagnosis, among women aged 25–64 years with at least 5 years of potential screening history

	Cases		Controls		OR
	*n*	%	*n*	%	(95% CI)
Stage I					
<1.5 years	22	11.5	345.3	36.1	0.14 (0.09–0.23)
1.5–2.5 years	14	7.3	153.3	16.0	0.20 (0.11–0.36)
2.5–3.5 years	18	9.4	97.0	10.1	0.41 (0.24–0.70)
3.5–5 years	23	12.0	112.9	11.8	0.45 (0.27–0.73)
>5 years	114	59.7	249.2	26.0	1
<3.5 years	54	28.3	595.6	62.2	0.20 (0.14–0.28)
Stage II+					
<1.5 years	16	8.8	299.1	33.4	0.10 (0.06–0.18)
1.5–2.5 years	10	5.5	148.2	16.6	0.13 (0.07–0.26)
2.5–3.5 years	5	2.8	102.4	11.4	0.10 (0.04–0.24)
3.5–5 years	13	7.2	77.1	8.6	0.33 (0.18–0.61)
>5 years	137	75.7	267.9	29.9	1
<3.5 years	31	17.1	549.7	61.4	0.11 (0.07–0.17)
All stages					
<1.5 years	40	9.8	707.0	34.5	0.12 (0.08–0.16)
1.5–2.5 years	25	6.1	328.6	16.1	0.16 (0.10–0.24)
2.5–3.5 years	25	6.1	215.9	10.6	0.24 (0.15–0.37)
3.5–5 years	40	9.8	219.9	10.7	0.37 (0.26–0.54)
>5 years	280	68.3	575.2	28.1	1
<3.5 years	90	22.0	1,251.6	61.1	0.15 (0.11–0.19)

NMHPVPR and virtual controls were used in this analysis.

Women who attended screening frequently (at least two screens a minimum of 10 months apart, with no interval >30 months between screens) were at significantly lower risk of both nonlocalized (OR = 0.10, 95% CI: 0.05–0.19) and Stage I cancer (OR = 0.43, 95% CI:0.28–0.65) than women who did not attend screening in a 5‐year period (Table 4). Women who attended screening in the previous 5 years, but did not meet the criteria for frequent screening (“infrequently” screened) were at significantly reduced risk of both nonlocalized (OR = 0.26, 95% CI: 0.18–0.37) and Stage I cancer (OR = 0.58, 95%CI: 0.40–0.82) compared to women not screened in 5 years, but at significantly greater risk of nonlocalized cancer compared to those screened frequently (OR = 2.54, 95% CI: 1.33–4.84). SA produced very similar results (Supporting Information Table S5). When restricted to women who attended screening in the 2.5 years prior to the date of index diagnosis or who had not attended in 5 years, the results were also very similar (Supporting Information Table S6).

**Table 4:** Odds ratios and 95% confidence intervals of cervical cancer for women who were frequently and infrequently screened by stage at diagnosis, among women aged 25–64 years with at least 5 years of potential screening history

	Cases		Controls		OR (95% CI)	OR (95% CI)
	N	%	N	%		
Stage I						
Frequently screened	41	21.5	301.8	31.5	0.43 (0.28–0.65)	1
Infrequently screened	80	41.9	435.3	45.5	0.58 (0.41–0.82)	1.35 (0.91–2.02)
Never screened	70	36.6	220.6	23.0	1	2.34 (1.54–3.54)
Stage II+						
Frequently screened	12	6.6	244.2	27.3	0.10 (0.05–0.19)	1
Infrequently screened	51	28.2	409.1	45.7	0.26 (0.18–0.37)	2.54 (1.33–4.84)
Never screened	118	65.2	241.4	27.0	1	9.95 (5.37–18.43)
All stages						
Frequently screened	56	13.7	590.3	28.8	0.23 (0.17–0.32)	1
Infrequently screened	145	35.4	947.0	46.3	0.37 (0.30–0.47)	1.61 (1.17–2.23)
Never screened	209	51.0	509.4	24.9	1	4.32 (3.16–5.92)

Women were considered regularly screened if they had at least two screens a minimum of 10 months apart, with no interval >30 months between screens, in the 5 years prior to diagnosis/pseudodiagnosis. NMHPVPR and virtual controls were used in this analysis.

When restricted to women who had only cytology screening (i.e., no HPV tests prior to diagnosis), the results of the three main analyses were very similar (Supporting Information Tables S7–S9).

## Discussion

Our study addressed three key relevant questions related to the performance of cervical screening. First, attending screening within a 3‐year period reduced the odds of nonlocalized cancer by 83%, and Stage I cancer by 48% compared to women not screened in 5 years. Second, women who had a negative screening test were at much lower risk of both nonlocalized and Stage I cancer for up to 5 years compared to women without a negative screen in the last 5 years, with a larger benefit in the first 3.5 years. Third, frequently attending cervical screening (at least two screens a minimum of 10 months apart, with no interval >30 months between screens) was associated with a 90% reduction in the odds of nonlocalized cervical cancer, and a 57% reduction in the odds of Stage I cervical cancer, compared to women who did not attend screening for 5 years. Notably, we found similar relative benefits of screening at ages 25–34, 35–49, 50–64 and aged ≥65 years for nonlocalized cancer.

It is important to acknowledge that cancers diagnosed before symptoms developed should be considered a success of cervical screening; 23% of cancers diagnosed at a known stage in New Mexico 2006–2016 were diagnosed at Stage IA. The stage distributions of cervical cancers diagnosed in New Mexico over the study time period including Stage IA were very similar to that computed for SEER18 registries overall (SEER*Stat November 2018; data not shown).

Women who were screened at least once every 2.5 years (frequently) had a relative risk of nonlocalized cancer of 0.39 compared to women screened infrequently. This was also the case when restricted to women who were screened within the 2.5 years prior to the date of index diagnosis, indicating that this is not purely due to the presence of a recent test, but to having had multiple tests in the 5‐year period. This was largely a study of cytology, with little co‐testing. The sensitivity of cytology for CIN2+ is around 71–75%;^21^ therefore, there is an advantage to having more frequent screenings, due to the high level of false negatives for a single cytology test. However, this does not mean that annual testing is an improvement, as demonstrated by the very similar risk of nonlocalized cancer 0–1.5 years after a negative screen compared to 2.5–3.5 years after a negative screen. On the contrary, while our study was not designed to assess the disadvantages of screening more frequently than current guidelines recommend, there are many reasons to dissuade this practice. First, more frequent screening increases the probability of having a false‐positive test (when either no precancerous lesion is present, or the precancerous lesion would regress without requiring intervention). Second, false‐positive tests have the potential to increase stress and anxiety if further diagnostic testing is required, in addition to the discomfort from a colposcopy. Additionally, there is the time and expense associated with unnecessary testing; in New Mexico, 28% of women who reside in rural areas must travel more than 30 minutes each‐way to seek diagnostic services.^22^

Recent guidelines recommend routine HPV cotesting in women aged 30–65 years.^4^ The majority of screening records in New Mexico in 2006–2016 were cytology tests taken alone, though the proportion of HPV tests or cotests increased with time (from 4.2% in 2006 to 54.7% in 2016), and when restricted to women aged 30–65 years, where cotests are routinely recommended, 67.8% were observed in 2016. Cotesting will increase the sensitivity of a single round of screening, and potentially support longer screening intervals *versus* intervals when screening by cytology alone.^23^ Whether longer screening intervals can be successfully adopted by the US in the absence of organized call‐recall systems should be given careful consideration. As cervical screening intervals lengthen for primary HPV testing and cotesting over time, it will be critical to monitor the proportion of women who fail to rescreen at 5‐year intervals. Although HPV‐based technologies are directed at improving screening efficiencies and reducing potential harms from screening, lengthening cervical cancer screening intervals in the US may not be readily implemented due to the lack of organized screening programs. Furthermore, the continuously changing landscape of cervical screening could result in an increase in cervical cancer incidence if women fail to return for screening or return beyond the duration of protection afforded.

While we have shown that cervical screening in New Mexico is effective at preventing cervical cancer, only 61% of controls aged 25–64 years had attended cervical screening in a 3‐year period. Therefore, initiatives which increase screening coverage are likely the best investment for improving the prevention of cervical cancer, especially among women from birth cohorts which did not benefit from HPV vaccination prior to sexual initiation. Since not all attendees return for their next screen, it is important to use the most sensitive screening test available.

Similar methods have been used to explore the effectiveness of cervical screening in Europe^8^, ^9^, ^10^, ^11^, ^24^, ^25^ and Australia.^26^ Andrae *et al*.^8^ found a slightly lower effect of screening in women aged 30–65 in Sweden for all stages (OR = 2.52) and Stage II+ (OR = 4.82), when considering women who were not screened compared to women who were screened in the recommended interval (3 yearly for women aged 30–50 and 5 yearly for women ages 50–60). Yang *et al*.^26^ found that even infrequent screening in Australia, defined as a pap test in only 1 year of a 4‐year period, was associated with an 85% reduction in risk of all stages of cervical cancer, and frequent screening (a pap in at least 2 years in a 4‐year period) was associated with around a 95% reduction in risk. These effects are slightly larger than those found for infrequently and frequent screening in our study, though our definition of frequent screening differs slightly.

New Mexico is the only state within the US where cervical screening data of this quality exist on a population basis, enabling the evaluation of cervical screening as practiced across a wide range of healthcare delivery settings. Screening recommendations and implementation approaches vary widely between countries,^27^ so results from one setting may not apply to another; for example, in the US, the vast majority of screening is opportunistic whereas in Sweden there is a national program where women are invited for screening.^28^ The importance of comprehensive audits of screening programs including the full target population is widely recognized.^29^, ^30^ Previous research on the effectiveness of cervical screening in the US has relied on data from women enrolled in health plans or integrated health systems^15^, ^16^ who may be at different risk of cervical cancer than the general population. Screening guidelines for the US have been almost exclusively based on the analysis of cervical screening data which are not representative of women and/or providers in the general population.^2^, ^31^ Furthermore, studies of cervical screening effectiveness in the US have been conducted in settings where screening is implemented by system‐specific screening guidelines. For example, Kaiser Permanente Northern California introduced HPV as part of a cotest in 2003,^32^ whereas HPV cotesting did not even begin utilization in mainstream clinical practice in New Mexico until 2013, following national cervical screening guidelines issued in 2012.^3^

It was not possible to select controls from a population register and link to their screening history. Only women who have attended screening at least once could be identified from the NMHPVPR; it was therefore important to augment this with virtual‐controls (who had not been screened since January 2006) based on the census. Had we not included virtual‐controls, we would have overestimated the impact of screening. We weighted the controls selected from the NMHPVPR by identifying the age‐specific proportion of matched women in the NMTR who had a screening record in the NMHPVPR. However, women who develop noncervical cancer may have different screening behaviors compared to the general population; we therefore reweighted the controls excluding women diagnosed with cancers which could have been screen‐detected (breast and colorectal), and the results were extremely similar (Supporting Information Fig. S1, Tables S3 and S5). Our results estimated 75% of controls aged 25–65 years had been screened in the past 5 years; this is consistent with previous investigations which estimated the 5‐year screening coverage for women aged 21–65 years in New Mexico to be around 80%.^19^

While we have not included any woman who we know to have had a hysterectomy, we only have incomplete information on hysterectomies (particularly prior to 2006). The situation is further complicated in that prior to 2012, the majority of women with a hysterectomy were still offered screening. If we add together the number of women in the screening registry with the number of women in New Mexico who have had a hysterectomy, the sum, in most age‐groups, is greater than the number in the census. Analyzing the data in this way would be equivalent to not allowing for unscreened (virtual) controls—it makes screening appear better than it is.

We have used the date of the first abnormal cytology or positive HPV within 5 months of diagnosis as the date of index diagnosis rather than the definitive date of diagnosis used by the NMTR,^33^ and considered screening in a 3‐ or 5‐year period prior to this date. We only have records of screening tests performed on women where addresses were recorded as a resident of New Mexico or which were taken from a New Mexico provider; whereas some women may have attended screening in other States which would have been missed. Some of the women selected as NMHPVPR controls may only have been resident in New Mexico for a limited period, for example, due to migration, therefore our data may not represent their full screening history since 2006. When limiting the analyses to women diagnosed with cervical cancer at age 25–64 years who had at least 5 years of screening history data, our sample was reduced to 410 cases. Screening guidelines varied both between and within organizations across the period of our study, so we could not evaluate the effect of screening among women who complied with screening guidelines. We have not linked HPV vaccination status to screening histories, but this is likely to have minimal impact on our results due to the long natural history from HPV infection to cervical cancer *versus* the introduction of HPV vaccination. We do not have sufficient women who were only screened using HPV testing to compare the effect of screening using cytology alone to those with HPV testing, nor sufficient numbers of women with adenocarcinomas who have at least 3 years of screening data when broken down by stage and screening history in order to investigate the effect of screening by histologic subtype.

In conclusion, our study demonstrates that routine screening at a population level has had a beneficial effect in preventing cervical cancer. However, only 61% of controls in our study had attended screening in a 3‐year period. Thus, increasing screening coverage will have the greatest impact in achieving further reductions in cervical cancer rates.

## Conflict of interest

J.C. and C.M.W. have received funds from grants, cooperative agreements or subcontracts related to cervical screening and triage through their institutions. J.C. reports grants to his institution and personal fees from Qiagen, Becton Dickinson (BD), Genera Biosystems (GB) and grants to his institution from Hologic, Gene First and Trovagene, all outside the submitted work. CMW reports receiving reagents and equipment for HPV genotyping, from Roche and GB through her institution and personal fees from BD all outside of the submitted work. R.L., P.D.S., C.M., C.L.W., M.R., Y.J.M., D.W.G. and I.C.S. have no interests to report.

## Supporting information


**Data S1**: Supporting Information

## Appendix 1

### 1.1

New Mexico HPV Pap Registry (NMHPVPR) Steering Committee Members:

Members of the New Mexico HPV Pap Registry (NMHPVPR) Steering Committee reviewed and gave input to the manuscript and supported the concept and directions of the NMHPVPR including the evaluations presented in this manuscript. The NMHPVPR Steering members participating in this effort are as follows: Nancy E. Joste, MD, University of New Mexico Health Sciences Center and Tricore Reference Laboratories, Albuquerque, New Mexico; Walter Kinney, MD, retired Kaiser Permanente Northern California; Cosette M. Wheeler, PhD, University of New Mexico Health Sciences Center; Ruth M. McDonald, MS, University of New Mexico Health Sciences Center; Michael Robertson, BS, University of New Mexico Health Sciences Center, Alan Waxman, MD MPH, University of New Mexico Health Sciences Center; Steven Jenison, MD, Community Member; Julia C. Gage, PhD, MPH, US National Cancer Institute; Philip E. Castle, PhD MPH, Albert Einstein School of Medicine; Vicki Benard, PhD, US Centers for Disease Control and Prevention; Debbie Saslow, PhD, American Cancer Society; Jane J. Kim PhD, Harvard TH Chan School of Public Health; Mark H. Stoler MD, University of Virginia; Jack Cuzick, PhD, Wolfson Institute of Preventive Medicine, London; and Giovanna Rossi Pressley, MSc, Collective Action Strategies; Kevin English, DrPh MPH, Albuquerque Area Southwest Tribal Epidemiology Center (AASTEC); No compensation was received for contributions to this manuscript by any named authors or by the NMHPVPR Steering Committee members.
