# Supplementary material for: Impact of screening on cervical cancer incidence: A population‐based case–control study in the United States
Source: Int J Cancer. 2019 Dec 31;147(3):887–96. doi: 10.1002/ijc.32826 (PMC7282928; doi:10.1002/ijc.32826)

Impact of screening on cervical cancer incidence. A population-based case-control study in the United States

Rebecca Landy PhD, Peter D. Sasieni PhD, Christopher Mathews BSc, Charles L. Wiggins PhD, Michael Robertson BS, Yolanda J. McDonald PhD, Daniel W. Goldberg PhD, Isabel C. Scarinci PhD, Jack Cuzick PhD, Cosette M. Wheeler PhD for the New Mexico HPV Pap Registry Steering Committee

Supplementary Methods p2

Supplementary Tables p4

Supplementary Figures p14

**Supplementary Methods**

SM1: Determining which tests are likely due to symptoms

Whether a test was taken as part of routine screening or in response to symptoms was unknown. Women diagnosed with cervical cancer are more likely to have a test in the months before diagnosis than a control woman is before her matched case’s diagnosis. To avoid bias, when considering whether women were screened in a given period, we excluded the months prior to the case’s diagnosis in which a higher proportion of cases were tested than controls. **Table S10** shows the proportion of cases and controls who had a cytology or HPV test recorded in the screening registry in the same month as diagnosis/pseudodiagnosis, and in each of the 6 months prior to diagnosis/pseudodiagnosis. At 5 months prior to diagnosis, the proportions of cases and controls in the screening registry become similar; therefore we take the date of diagnosis as the earliest date of an abnormal cytology or positive HPV test taken within the 5 months prior to diagnosis and the date of histological diagnosis.

SM2: Weighting of controls

The fractional number of virtual controls was determined by the proportions of a second set of controls selected from the NMTR that were not in the NMHPVPR. The fractional number of controls was obtained by weighting a single virtual-control. The controls used to determine the weights were women diagnosed with a non-cervical cancer after the case’s date of diagnosis, with a limit of one control with non-melanoma skin cancer per case. Six controls per case were matched on date of birth; 2 of the 6 controls were additionally matched on county. Additional details on matching are provided in **SM3.** Women were not eligible as NMTR controls if they were known to have had a hysterectomy prior to the case’s diagnosis. NMTR control women’s screening histories were extracted from the NMHPVPR. Any woman in the NMTR who was not linked to the NMHPVPR was considered not to have attended for cervical screening in New Mexico since 2006. Overall, 4022 of the 5186 (77.6%) NMTR controls had a cytology or HPV test recorded in the NMHPVPR.

We estimated the proportion of women in each age group *j* who did not have a record in the NMHPVPR in the years 2006-2016 (*p_j_*). Each virtual (unscreened) control was assigned weight 5*p_j_*, and each screened control was assigned weight (1- *p_j_*). We estimated *p_j_* as the proportion of the NMTR controls in each 5-year age group who had a screening record in the NMHPVPR. For sensitivity analyses, to avoid over-representation of women with frequent screening, we excluded women who were diagnosed with breast or colorectal cancer from the NMTR controls, in case these women were diagnosed with screen-detected cancers, and in case these women had different screening histories versus the general population.

SM3: Matching cases and controls on census tract of residence

All cases and controls were matched using the census tract of residence at diagnosis for cases and at screening for controls, however the quality of this information varied. An exact street address was recorded for most women, but sometimes only a P.O. Box or zip code was known. In these instances, the census tract is based on the centroid of the zip code. Therefore, women with a P.O. Box were matched to women in the same census tract as the P.O. Box. We include sensitivity analyses excluding cases whose address was a P.O. Box or zip code.

SM4: Sensitivity analyses, results

As the controls were matched to the cases on census tract, adjustment for census-tract level sociodemographic variables made no difference (SA1), nor did excluding women who had a P.O. Box or zip code as their address (SA2). The results were extremely similar for the two sets of weights (SA3). SA4, which excluded the virtual-controls from the analyses, always overestimated the effect of screening. There was slightly less effect of screening when all ages were considered (SA5), and no difference when the age range was extended to 25-69y (SA6). Finally, there was slightly less effect when the reference category was women who had not attended screening in the past 3 years compared to not attending for 5 years (SA7).

Supplementary Table 1: Census-tract level sociodemographic variables used in the sensitivity analyses

| variable label | variable description | by age? | by sex? | Source of data |
| --- | --- | --- | --- | --- |
| Urban | the proportion of the population who lived in urban areas | no | No | Census 2010 Summary File 1 - P2 |
| High school graduate | the proportion of the population who had graduated high school | yes | Yes | ACS 2015 5-year estimates – B15001 |
| Bachelors degree | the proportion of the population who had a bachelor's degree or higher | yes | Yes | ACS 2015 5-year estimates – B15001 |
| Unemployment rate | the proportion of the population aged 16+ who were unemployed | yes | Yes | ACS 2015 5-year estimates – B23001 |
| Poor English | (the proportion of households which have no-one aged 14+ who speaks English very well | no | No | ACS 2015 5-year estimates - S1602 |
| Insurance status | the proportion of the population who were uninsured | yes | Yes | ACS 2015 5-year estimates - B27001 |
| Poverty | the proportion of people who were below the poverty level | no | No | ACS 2015 5-year estimates - S1701 |
| Hispanic | the proportion of the population who were Hispanic | yes | Yes | Census 2010 Summary File 1 - P12H |
| Native American | the proportion of the population who were Native American | yes | Yes | Census 2010 Summary File 1 - P12C |
| Black | the proportion of the population who were Black | yes | Yes | Census 2010 Summary File 1 - P12B |
| White - non Hispanic | the proportion of the population who were white | yes | Yes | Census 2010 Summary File 1 - P12H |
| Other ethnicity | the proportion of the population who were not non-Hispanic White, Black, Hispanic or Native American | yes | Yes | Census 2010 Summary File 1 - P12B, P12C, P12H |

Supplementary Table 2: Odds ratios and 95% confidence intervals of cervical cancer by screening attendance and stage at diagnosis, among women aged 25-64 years with at least 3 years of potential screening history

|  | Cases | | Controls | | OR (95%CI) | OR (95%CI) |
| --- | --- | --- | --- | --- | --- | --- |
|  | N | % | N | % |  |  |
| **Stage 1A** |  |  |  | | | |
| screened in the last 3 years | 70 | 59.3 | 367.3 | 62.5 | 0.78 (0.48-1.28) | 1 |
| screened in the last 5 years, but not the last 3 years* | 22 | 18.6 | 114.0 | 19.4 | 0.79 (0.43-1.47) | 1.01 (0.60-1.70) |
| not screened in the last 5 years, with ≥5 years of potential screening data | 26 | 22.0 | 106.8 | 18.2 | 1 | 1.28 (0.78-2.08) |
| **Stage 1B** |  |  |  |  |  |  |
| screened in the last 3 years | 50 | 47.2 | 344.5 | 64.6 | 0.38 (0.24-0.62) | 1 |
| screened in the last 5 years, but not the last 3 years* | 22 | 20.8 | 99.5 | 18.6 | 0.58 (0.32-1.06) | 1.52 (0.88-2.62) |
| not screened in the last 5 years, with ≥5 years of potential screening data | 34 | 32.1 | 89.7 | 16.8 | 1 | 2.61 (1.61-4.24) |
| **Stage 2** |  |  |  |  |  |  |
| screened in the last 3 years | 17 | 33.3 | 150.7 | 59.9 | 0.22 (0.11-0.43) | 1 |
| screened in the last 5 years, but not the last 3 years* | 6 | 11.8 | 46.1 | 18.3 | 0.25 (0.10-0.66) | 1.15 (0.43-3.08) |
| not screened in the last 5 years, with ≥5 years of potential screening data | 28 | 54.9 | 54.6 | 21.7 | 1 | 4.55 (2.33-8.89) |
| **Stage 3+** |  |  |  |  |  |  |
| screened in the last 3 years | 40 | 22.5 | 530.1 | 60.1 | 0.16 (0.10-0.23) | 1 |
| screened in the last 5 years, but not the last 3 years* | 48 | 27.0 | 165.1 | 18.7 | 0.60 (0.40-0.90) | 3.85 (2.45-6.05) |
| not screened in the last 5 years, with ≥5 years of potential screening data | 90 | 50.6 | 186.7 | 21.2 | 1 | 6.39 (4.27-9.56) |

* or not screened in the last 3 years with <5 years of potential screening data.

NMHPVPR and virtual controls were used in this analysis.

Supplementary Table 3: Odds ratios and 95% confidence intervals of cervical cancer by time since last negative screen and stage at diagnosis, among women aged 25-64 years with at least 5 years of potential screening history, a) using alternative weights and b) adjusting for sociodemographic variables

|  | OR (95% CI) using alternative weights | OR (95% CI) adjusting for sociodemographic variables |
| --- | --- | --- |
|  |  |  |
| **Stage 1** |  |  |
| <1.5 years | 0.13 (0.07-0.25) | 0.13 (0.07-0.24) |
| 1.5-2.5 years | 0.27 (0.14-0.53) | 0.26 (0.13-0.51) |
| 2.5-3.5 years | 0.21 (0.08-0.56) | 0.20 (0.08-0.53) |
| 3.5-5 years | 0.44 (0.24-0.84) | 0.44 (0.23-0.83) |
| >5 years | 1 | 1 |
| <3.5 years | 0.18 (0.11-0.29) | 0.18 (0.11-0.28) |
|  |  |  |
| **Stage 2+** |  |  |
| <1.5 years | 0.10 (0.06-0.18) | 0.10 (0.06-0.18) |
| 1.5-2.5 years | 0.13 (0.07-0.26) | 0.13 (0.07-0.26) |
| 2.5-3.5 years | 0.09 (0.04-0.24) | 0.10 (0.04-0.24) |
| 3.5-5 years | 0.33 (0.18-0.61) | 0.32 (0.18-0.59) |
| >5 years | 1 | 1 |
| <3.5 years | 0.11 (0.07-0.17) | 0.11 (0.07-0.16) |
|  |  |  |
| **All stages** |  |  |
| <1.5 years | 0.12 (0.08-0.16) | 0.11 (0.08-0.16) |
| 1.5-2.5 years | 0.16 (0.10-0.24) | 0.15 (0.10-0.23) |
| 2.5-3.5 years | 0.24 (0.15-0.37) | 0.23 (0.15-0.36) |
| 3.5-5 years | 0.37 (0.26-0.53) | 0.37 (0.26-0.52) |
| >5 years | 1 | 1 |
| <3.5 years | 0.15 (0.11-0.19) | 0.14 (0.11-0.19) |

NMHPVPR and virtual controls were used in this analysis.

Supplementary Table 4: Odds ratios and 95% confidence intervals of cervical cancer by time since last negative screen and stage at diagnosis, among women aged 25-64 years with at least 3 years of potential screening history

|  | Cases | | Controls | | unadjusted OR |
| --- | --- | --- | --- | --- | --- |
|  | N | % | N | % | (95% CI) |
| **Stage 1** |  |  |  |  |  |
| <1.5 years | 27 | 10.8 | 441.1 | 35.4 | 0.13 (0.09-0.21) |
| 1.5-2.5 years | 22 | 8.8 | 191.2 | 15.3 | 0.25 (0.15-0.41) |
| 2.5-3.5 years | 24 | 9.6 | 133.3 | 10.7 | 0.39 (0.24-0.64) |
| 3.5-5 years | 23 | 9.2 | 118.8 | 9.5 | 0.42 (0.26-0.69) |
| no negative, <5 yrs potential data | 39 | 15.7 | 113.1 | 9.1 | 0.75 (0.50-1.15) |
| >5 years | 114 | 45.8 | 249.2 | 20.0 | 1 |
| *<3.5 years* | *73* | *29.3* | *765.5* | *61.4* | *0.21 (0.15-0.29)* |
| 3.5-5 years, or <5 yrs potential data | 62 | 24.9 | 231.9 | 18.6 | 0.58 (0.41-0.83) |
| **Stage 2+** |  |  |  |  |  |
| <1.5 years | 22 | 9.6 | 392.7 | 34.7 | 0.11 (0.07-0.18) |
| 1.5-2.5 years | 11 | 4.8 | 172.0 | 15.2 | 0.13 (0.07-0.24) |
| 2.5-3.5 years | 7 | 3.1 | 124.3 | 11.0 | 0.11 (0.05-0.24) |
| 3.5-5 years | 14 | 6.1 | 84.7 | 7.5 | 0.32 (0.18-0.59) |
| no negative, <5 yrs potential data | 38 | 16.6 | 91.6 | 8.1 | 0.81 (0.53-1.24) |
| >5 years | 137 | 59.8 | 267.9 | 23.6 | 1 |
| *<3.5 years* | *40* | *17.5* | *689.0* | *60.8* | *0.11 (0.08-0.17)* |
| 3.5-5 years, or <5 yrs potential data | 52 | 22.7 | 176.3 | 15.6 | 0.58 (0.40-0.83) |
| **All stages** |  |  |  |  |  |
| <1.5 years | 52 | 9.9 | 914.8 | 34.8 | 0.12 (0.09-0.16) |
| 1.5-2.5 years | 35 | 6.6 | 399.8 | 15.2 | 0.18 (0.12-0.26) |
| 2.5-3.5 years | 33 | 6.3 | 280.1 | 10.7 | 0.24 (0.16-0.36) |
| 3.5-5 years | 41 | 7.8 | 234.3 | 8.9 | 0.36 (0.25-0.51) |
| no negative, <5 yrs potential data | 86 | 16.3 | 225.0 | 8.6 | 0.79 (0.59-1.04) |
| >5 years | 280 | 53.1 | 575.2 | 21.9 | 1 |
| *<3.5 years* | *120* | *22.8* | *1594.8* | *60.7* | *0.15 (0.12-0.19)* |
| 3.5-5 years, or <5 yrs potential data | 127 | 24.1 | 459.4 | 17.5 | 0.57 (0.45-0.72) |

NMHPVPR and virtual controls were used in this analysis

Supplementary Table 5: Odds ratios and 95% confidence intervals of cervical cancer for women who were frequently and infrequently screened by stage at diagnosis, among women aged 25-64 years with at least 5 years of potential screening history; sensitivity analyses a) using alternative weights and b) adjusting for sociodemographic variables

|  | OR (95% CI) using alternative weights | | OR (95% CI) adjusting for sociodemographic variables | |
| --- | --- | --- | --- | --- |
| **Stage 1** |  |  |  |  |
| Frequently screened | 0.43 (0.28-0.65) | 1 | 0.42 (0.28-0.63) | 1 |
| Infrequently screened | 0.58 (0.41-0.82) | 1.35 (0.90-2.01) | 0.57 (0.40-0.82) | 1.37 (0.92-2.05) |
| Never screened | 1 | 2.34 (1.54-3.55) | 1 | 2.39 (1.58-3.61) |
| **Stage 2+** |  |  |  |  |
| Frequently screened | 0.10 (0.05-0.18) | 1 | 0.10 (0.05-0.18) | 1 |
| Infrequently screened | 0.25 (0.18-0.36) | 2.54 (1.33-4.84) | 0.25 (0.17-0.35) | 2.56 (1.33-4.92) |
| Never screened | 1 | 10.03 (5.41-18.58) | 1 | 10.32 (5.49-19.39) |
| **All stages** |  |  |  |  |
| Frequently screened | 0.23 (0.17-0.32) | 1 | 0.22 (0.16-0.31) | 1 |
| Infrequently screened | 0.37 (0.29-0.47) | 1.61 (1.17-2.23) | 0.36 (0.29-0.46) | 1.63 (1.18-2.26) |
| Never screened | 1 | 4.35 (3.17-5.95) | 1 | 4.48 (3.27-6.13) |

Analyses using alternative weights and analyses adjusted for sociodemographic variables, see Supplementary Materials 2.

NMHPVPR and virtual controls were used in this analysis.

Supplementary Table 6: Odds ratios and 95% confidence intervals of cervical cancer for women who were frequently and infrequently screened by stage at diagnosis, among women aged 25-64 years with at least 5 years of potential screening history, restricted to women with a screening test in the past 2.5 years, or with no screening tests in the past 5 years.

|  | Cases | | Controls | | OR (95% CI) | OR (95% CI) |
| --- | --- | --- | --- | --- | --- | --- |
|  | N | % |  |  |  |  |
| **Stage 1** |  | |  | | |  |
| Frequently screened | 40 | 25.6 | 287.8 | 38.5 | 0.44 (0.29-0.67) | 1 |
| Infrequently screened | 46 | 29.5 | 238.8 | 32.0 | 0.61 (0.40-0.91) | 1.39 (0.88-2.18) |
| Never screened | 70 | 44.9 | 220.6 | 29.5 | 1 | 2.28 (1.50-3.48) |
| **Stage 2+** |  | |  | | |  |
| Frequently screened | 11 | 7.0 | 233.8 | 32.8 | 0.10 (0.05-0.18) | 1 |
| Infrequently screened | 29 | 18.4 | 237.8 | 33.4 | 0.25 (0.16-0.39) | 2.59 (1.27-5.29) |
| Never screened | 118 | 74.7 | 241.4 | 33.9 | 1 | 10.39 (5.48-19.71) |
| **All stages** |  | |  | | |  |
| Frequently screened | 54 | 15.6 | 563.2 | 35.1 | 0.23 (0.17-0.32) | 1 |
| Infrequently screened | 84 | 24.2 | 531.9 | 33.2 | 0.38 (0.29-0.51) | 1.65 (1.15-2.36) |
| Never screened | 209 | 60.2 | 509.4 | 31.7 | 1 | 4.28 (3.11-5.89) |

NMHPVPR and virtual controls were used in this analysis.

Supplementary Table 7: Odds ratios and 95% confidence intervals of cervical cancer by screening attendance and stage at diagnosis, among women aged 25-64 years with at least 3 years of potential screening history, excluding women with any HPV tests prior to diagnosis

|  | Cases | | Controls | | OR (95% CI) | OR (95% CI) |
| --- | --- | --- | --- | --- | --- | --- |
|  | N | % | N | % |  |  |
| **Stage 1** |  |  |  | | | |
| screened in the last 3 years | 65 | 37.4 | 353.6 | 54.1 | 0.38 (0.26-0.56) | 1 |
| screened in the last 5 years, but not the last 3 years* | 45 | 25.9 | 167.0 | 25.6 | 0.56 (0.36-0.86) | 1.47 (0.97-2.23) |
| not screened in the last 5 years, with ≥5 years of potential screening data | 64 | 36.8 | 132.5 | 20.3 | 1 | 2.63 (1.78-3.88) |
| **Stage 2+** |  |  |  |  |  |  |
| screened in the last 3 years | 35 | 17.6 | 363.8 | 49.5 | 0.17 (0.11-0.26) | 1 |
| screened in the last 5 years, but not the last 3 years* | 49 | 24.6 | 166.9 | 22.7 | 0.52 (0.36-0.77) | 3.05 (1.91-4.87) |
| not screened in the last 5 years, with ≥5 years of potential screening data | 115 | 57.8 | 204.7 | 27.8 | 1 | 5.84 (3.87-8.80) |
| **All Stages** |  |  |  |  |  |  |
| screened in the last 3 years | 103 | 25.2 | 772.6 | 50.9 | 0.25 (0.19-0.33) | 1 |
| screened in the last 5 years, but not the last 3 years* | 106 | 26.0 | 372.1 | 24.5 | 0.54 (0.41-0.70) | 2.14 (1.59-2.87) |
| not screened in the last 5 years, with ≥5 years of potential screening data | 199 | 48.8 | 373.9 | 24.6 | 1 | 3.99 (3.07-5.20) |

* or not screened in the last 3 years with <5 years of potential screening data.

NMHPVPR and virtual controls were used in this analysis

Supplementary Table 8: Odds ratios and 95% confidence intervals of cervical cancer by time since last negative screen and stage at diagnosis, among women aged 25-64 years with at least 5 years of potential screening history, excluding women with any HPV tests prior to diagnosis

|  | Cases | | Controls | | OR |
| --- | --- | --- | --- | --- | --- |
|  | N | % | N | % | (95% CI) |
| **Stage 1** |  |  |  |  |  |
| <1.5 years | 14 | 11.5 | 120 | 28.6 | 0.20 (0.11-0.36) |
| 1.5-2.5 years | 4 | 3.3 | 73 | 17.3 | 0.09 (0.03-0.26) |
| 2.5-3.5 years | 11 | 9.0 | 42 | 9.9 | 0.44 (0.22-0.90) |
| 3.5-5 years | 12 | 9.8 | 50 | 11.9 | 0.40 (0.20-0.79) |
| >5 years | 81 | 66.4 | 136 | 32.3 | 1 |
| *<3.5 years* | *29* | *23.8* | *235* | *55.8* | *0.21 (0.13-0.33)* |
|  |  |  |  |  |  |
| **Stage 2+** |  |  |  |  |  |
| <1.5 years | 13 | 8.4 | 133 | 25.0 | 0.16 (0.09-0.30) |
| 1.5-2.5 years | 6 | 3.9 | 83 | 15.5 | 0.12 (0.05-0.28) |
| 2.5-3.5 years | 3 | 1.9 | 57 | 10.8 | 0.09 (0.03-0.28) |
| 3.5-5 years | 8 | 5.2 | 50 | 9.5 | 0.26 (0.12-0.57) |
| >5 years | 125 | 80.6 | 208 | 39.2 | 1 |
| *<3.5 years* | *22* | *14.2* | *273* | *51.3* | *0.13 (0.08-0.22)* |
|  |  |  |  |  |  |
| **All stages** |  |  |  |  |  |
| <1.5 years | 28 | 8.2 | 275 | 26.5 | 0.17 (0.11-0.26) |
| 1.5-2.5 years | 10 | 2.9 | 162 | 15.6 | 0.10 (0.05-0.20) |
| 2.5-3.5 years | 15 | 4.4 | 108 | 10.4 | 0.24 (0.13-0.41) |
| 3.5-5 years | 23 | 6.7 | 113 | 10.9 | 0.34 (0.22-0.55) |
| >5 years | 266 | 77.8 | 383 | 36.8 | 1 |
| *<3.5 years* | *53* | *15.5* | *545* | *52.4* | *0.16 (0.2-0.23)* |

NMHPVPR and virtual controls were used in this analysis.

Supplementary Table 9: Odds ratios and 95% confidence intervals of cervical cancer for women who were frequently and infrequently screened by stage at diagnosis, among women aged 25-64 years with at least 5 years of potential screening history, excluding women with any HPV tests prior to diagnosis

|  | Cases | | Controls | | OR (95% CI) | OR (95% CI) |
| --- | --- | --- | --- | --- | --- | --- |
|  | N | % | N | % |  |  |
| **Stage 1** |  | |  | | |  |
| Frequently screened | 12 | 9.8 | 103.8 | 24.7 | 0.24 (0.12-0.46) | 1 |
| Infrequently screened | 44 | 36.1 | 182.0 | 43.2 | 0.49 (0.32-0.76) | 2.09 (1.06-4.12) |
| Never screened | 66 | 54.1 | 135.1 | 32.1 | 1 | 4.23 (2.19-8.16) |
| **Stage 2+** |  | |  | | |  |
| Frequently screened | 5 | 3.2 | 106.7 | 20.1 | 0.08 (0.03-0.21) | 1 |
| Infrequently screened | 35 | 22.6 | 219.2 | 41.2 | 0.29 (0.19-0.43) | 3.41 (1.30-8.91) |
| Never screened | 115 | 74.2 | 205.6 | 38.7 | 1 | 11.93 (4.75-29.99) |
| **All stages** |  | |  | | |  |
| Frequently screened | 17 | 5.6 | 223.3 | 21.5 | 0.14 (0.09-0.24) | 1 |
| Infrequently screened | 84 | 27.8 | 439.6 | 42.3 | 0.36 (0.27-0.48) | 2.51 (1.46-4.32) |
| Never screened | 201 | 66.6 | 377.5 | 36.3 | 1 | 7.00 (4.17-11.74) |

Women were considered frequently screened if they had at least 2 screens a minimum of 10 months apart, with no interval >30 months between screens, in the 5 years prior to diagnosis/pseudo-diagnosis. NMHPVPR and virtual controls were used in this analysis

Supplementary Table 10: The percentage of cases and controls screened in each of the 6 months prior to the matched case's date of diagnosis

| Number of months prior to diagnosis/pseudo-diagnosis (X) | N cases 'screened' within X months of diagnosis | N cancer registry controls 'screened' within X months of diagnosis | % cases screened X months before diagnosis | % of cancer registry controls screened X months before diagnosis |
| --- | --- | --- | --- | --- |
| 0 | 221 | 118 | 25.2% | 2.6% |
| 1 | 162 | 107 | 18.5% | 2.3% |
| 2 | 87 | 126 | 9.9% | 2.7% |
| 3 | 45 | 104 | 5.1% | 2.3% |
| 4 | 36 | 118 | 4.1% | 2.6% |
| 5 | 22 | 110 | **2.5%** | **2.4%** |
| 6 | 18 | 117 | 2.1% | 2.5% |

A woman is defined to be screened if she had an HPV or cytology test

Supplementary Figure 1: Odds ratios and 95% confidence intervals for risk of cervical cancer by stage for women screened within the last 3 years compared to women not screened in the last 5 years, restricted to women with ≥40 months of screening history; sensitivity analyses


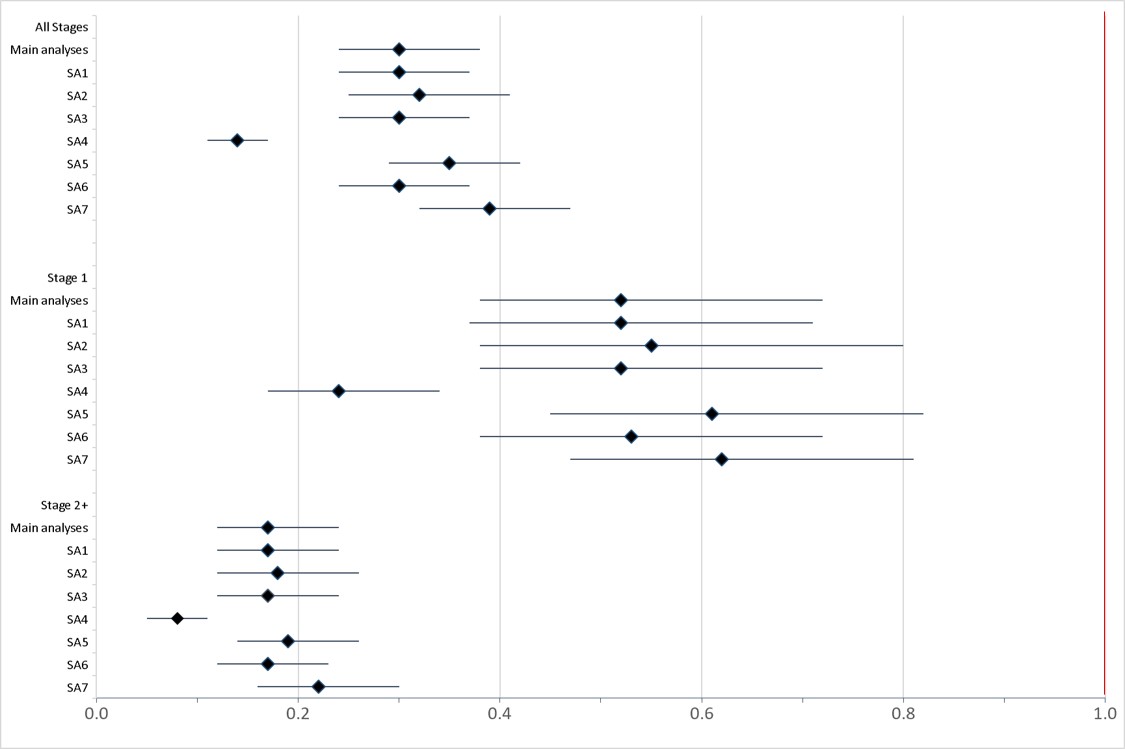


Footnote: SA1: adjusted for the census-tract level sociodemographic variables given in Table S1. SA2: excluded women whose address was a P.O. Box or zip code (see SM3). SA3: alternative weights (see SM2). SA4: excluded virtual (unscreened) controls from the analysis. SA5: included women of all ages. SA6: included women aged 25-69y. SA7: used a reference category of women who had not attended screening in a 3-year period.

Supplementary Figure 2: Odds ratios and 95% confidence intervals for risk of advanced stage cervical cancer within 3 years of a negative test, compared to women who have not had a negative test within the last 5 years, restricted to women with ≥5 years of screening history


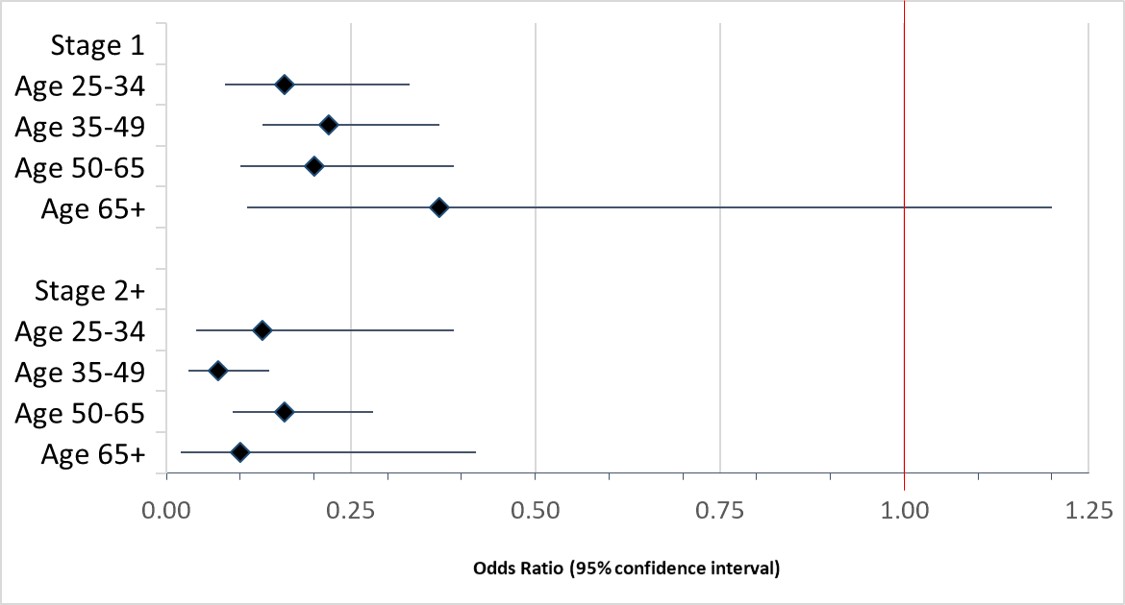

Supplement: Supplementary file 1 — Data S1: Supporting Information [file IJC-147-887-s001.docx]
